# The hidden link between circadian entropy and mental health disorders

**DOI:** 10.1038/s41398-022-02028-3

**Published:** 2022-07-14

**Authors:** Amal Alachkar, Justine Lee, Kalyani Asthana, Roudabeh Vakil Monfared, Jiaqi Chen, Sammy Alhassen, Muntaha Samad, Marcelo Wood, Emeran A. Mayer, Pierre Baldi

**Affiliations:** 1 Department of Pharmaceutical Sciences, School of Pharmacy and Pharmaceutical Sciences, University of California, Irvine, CA USA; 2 Institute for Genomics and Bioinformatics, University of California, Irvine, CA USA; 3 Center for the Neurobiology of Learning and Memory, University of California, Irvine, CA USA; 4 Department of Computer Science, School of Information and Computer Sciences, University of California, Irvine, CA USA; 5 Department of Neurobiology and Behavior, School of Biological Sciences, University of California, Irvine, CA USA; 6 G. Oppenheimer Center of Neurobiology of Stress & Resilience and Goldman Luskin Microbiome Center, Vatche and Tamar Manoukian Division of Digestive Diseases, University of California, Los Angeles, CA USA

**Keywords:** Neuroscience, Diseases

## Abstract

The high overlapping nature of various features across multiple mental health disorders suggests the existence of common psychopathology factor(s) (p-factors) that mediate similar phenotypic presentations across distinct but relatable disorders. In this perspective, we argue that circadian rhythm disruption (CRD) is a common underlying p-factor that bridges across mental health disorders within their age and sex contexts. We present and analyze evidence from the literature for the critical roles circadian rhythmicity plays in regulating mental, emotional, and behavioral functions throughout the lifespan. A review of the literature shows that coarse CRD, such as sleep disruption, is prevalent in all mental health disorders at the level of etiological and pathophysiological mechanisms and clinical phenotypical manifestations. Finally, we discuss the subtle interplay of CRD with sex in relation to these disorders across different stages of life. Our perspective highlights the need to shift investigations towards molecular levels, for instance, by using spatiotemporal circadian “omic” studies in animal models to identify the complex and causal relationships between CRD and mental health disorders.

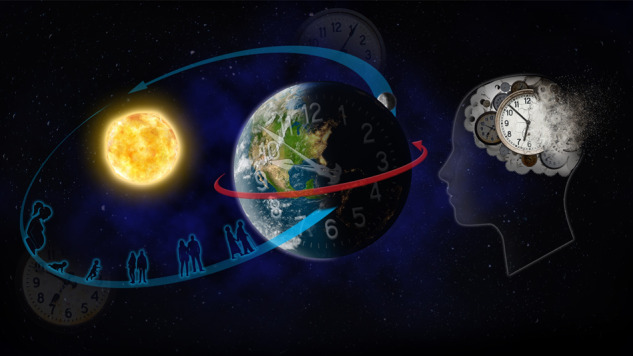

## Introduction

The existence of a high overlap of features across multiple mental health disorders [1, 2]. suggests the presence of common psychopathology factors (p-factors) that mediate similar phenotypic presentations across these disorders [3]. One hypothesis posits that every mental health disorder predisposes a patient to every other mental health disorder [2]. Thus, identifying these factors could provide a foundation for linking these disorders to this predisposition phenomenon [1]. We propose that circadian rhythmicity disruption (CRD) is one underlying biological p-factor shared by most mental health disorders, including neurodevelopmental, and aging-related mental disorders. Box 1 and 2.

Circadian rhythmicity is a fundamental biological feature that has recently garnered greater attention in neuroscience and psychiatry [4]. Circadian rhythms are oscillating patterns of physiological activity, which temporally regulate biological processes in one solar day. Synchronization of the biological rhythm to the 24 h light-dark cycle is mediated by an intrinsic sensitivity to light, which couples biological functions to different diurnal phases. Sleep-wake cycles, hormonal production and release, and body temperature control are highly regulated processes under the tight control of the circadian rhythm. Effective operation of these endogenous time-keeping mechanisms is necessary for the survival of most living organisms, underscoring the highly conserved nature of circadian systems. Unsurprisingly, normal circadian rhythmicity plays a critical role in neurodevelopment and cognitive function in sex-and age-dependent manners. This is partly due to circadian mechanisms’ sensitivity to hormonal signals like estrogen and cortisol. Consequently, CRD or circadian timing signals’ desynchrony may impact neurodevelopmental and mental outcomes.

In this perspective, we propose that disruption of circadian rhythmicity is a common underlying p-factor mediating these mental health disorders and argue for dynamic consequences of CRD in the context of age (life rhythm) with respect to neurodevelopmental disorders, adolescence and midlife psychiatric disorders, and aging-related neurodegenerative disorders. In the following sections, we review evidence of circadian disruption in the context of these disorders and explore evidence of the dynamic consequences of circadian disruption in the context of age and its interaction with sex in these disorders.

Box 1 Burdens of mental disorders Mental disorders, from neurodevelopmental to neurodegenerative, are the leading cause of disability worldwide. Collectively, they comprise approximately 13% of the global burden of disease [176] (Fig. 4). Together, it has been estimated that they will cost nations, families, and affected individuals approximately $6 trillion by 2030, though the cost burden varies among the disorders. For example, in the U.S., Alzheimer’s disease (AD) assumes a tremendous financial burden to society, costing an estimated $305 billion in 2020 alone [158]. Furthermore, as the demographic of Americans aged 65 and older is growing, the annual number of new cases of AD and other aging-related mental health disorders is projected to double by 2050 [177]. Consequently, total healthcare costs for AD treatment are expected to increase to more than $1 trillion as the population ages [178].

Along with the prevalence and cost trend for aging-related mental disorders, reports show a steady increase in the number of children diagnosed with neurodevelopmental disorders, namely attention-deficit/hyperactivity disorder (ADHD) or autism spectrum disorders (ASD) [179]. Neurodevelopmental disorders generate a similarly crushing financial burden, with an estimated cost of $268 billion and $266 billion for ASD and ADHD, respectively [159, 180] (Fig. 4), and for ASD are expected to rise to $461 billion by 2025 [159]. Unfortunately, the exact prevalence of neurodevelopmental disorders can be higher, as many children go undiagnosed, especially with respect to another prominent neurodevelopmental disorder—Tourette syndrome (TS)—implying that the aforementioned neurodevelopmental disorder-related costs are likely underestimated. According to the Center for Disease Control (CDC), one in 6 children between the ages of 3 and 17 were reported as being diagnosed with a neurodevelopmental disorder [163] (Fig. 5), with approximately 6.1 million children diagnosed with ADHD [164]. However, about half of the children with TS are never formally diagnosed with the disorder. Studies have suggested that about 1 in every 162 children younger than 17 years could be diagnosed with TS. On the other hand, some mental health disorders associated with late adolescence and early adulthood, such as schizophrenia (SCZ) and bipolar disorder (BD), show a relatively low prevalence; however, the costs to society remain considerably high. Annual SCZ-related costs have been estimated to range between $94 million and $102 billion in the U.S., with indirect costs (i.e., productivity losses due to morbidity and premature mortality) contributing to approximately 50–80% [160] of the total (Fig. 4). For BD, the direct and indirect cost was $45 billion in 1991 [181, 182]. More common adolescence and adulthood disorders, such as major depressive disorder (MDD) and anxiety disorders (AXDs), show a much higher prevalence and lower associated financial burden (Figs. 4 & 5). MDD creates a total annual economic burden of an estimated $210.5 million [161], with a direct-to-indirect cost ratio of 1:1.9, according to the American Psychiatric Association. Indeed, the prevalence of MDD is higher than BD, with around 16.1 million American adults (6.7%) aged 18 or over being diagnosed with MDD annually [165]. AXDs are the most prevalent mental health disorders among Americans, affecting about 19% of the population [162, 166]. However, only 36.9% receive treatment [183], explaining the lower associated societal costs.

Box 2 The sex-dependent and overlapping nature of mental disorders The shared clinical features and neuropathological mechanisms across many mental disorders suggest that disorders’ distinguishing characteristics may be due to normal physiological variation rather than being intrinsic attributes of the disorder per se. For example, according to the Center for Disease Control (CDC), six out of 10 children with ADHD had at least one other co-occurring neurodevelopmental disorder (NDD) [164], and 86% with TS were also diagnosed with at least one other mental disorder [167]. In addition, evidence regarding apparent sex-specific biases among several mental disorders implies the presence of pathogenic mechanisms that are sensitive to biological sex differences. NDDs are notorious for exhibiting sex-dependent expression. For instance, the male-to-female ratio for both attention-deficit/hyperactivity disorder (ADHD) and Tourette syndrome (TS) is 4:1, and 3:1 for autism spectrum disorders (ASD) [140, 169–171] (Fig. 6).

On the other hand, female populations are more prone to age-related disorders, with a male-to-female ratio of around 2:3 for AD [172]. Similarly, the prevalence of major depressive disorder (MDD), an affective disorder associated with adolescence and adulthood, is two-fold higher in females [173] (Fig. 6). Females are also more likely to develop anxiety disorders (AXDs) than males, with a prevalence of 30.5% to 19.2%, respectively [174]. Thus, in the context of these disorders the roles of sex-dependent processes regarding age are worth investigating. This would allow for the examination of potential risks or protections afforded by sex-specific physiology. Further, according to the Center for Disease Control (CDC), six out of 10 children with ADHD had at least one other co-occurring NDD [164] and 86% with TS were also diagnosed with at least one other mental disorder [167]. At the etiological level, schizophrenia (SCZ) and ASD have repeatedly shared several genetic factors. Symptomatically, socio-cognitive deficits and emotional dysregulation are core features across several disorders, such as AXD, ASD, SCZ, BD, and MDD.

Furthermore, MDD clinical presentations often resemble depressive episodes of BD, suggesting common etiopathological mechanisms. Not surprisingly, current definitions of mental disorders are frequently questioned; additionally, there is widespread suspicion that a greater degree of continuity exists across mental disorders.

## Overview of circadian rhythm regulation and dysregulation

Circadian rhythms are generated through oscillating patterns of physiological activity and couple biological functionality to the 24 h light-dark cycle. Intrinsic sensitivity to light primarily mediates this synchronization. Light-dark cues modulate circadian rhythms by initiating internal signaling cascades, which involve several endogenously formed molecules, including proteins and neurotransmitters. Such molecules can then generate signaling cascades of their own, which may amplify the initial light-dark cue. However, due to the involvement of a wide array of signaling cascades and their respective components, there are many points along this transduction pathway where signals can be interrupted or altered, resulting in some downstream consequences of this disruption.

An explicit example of this relationship is observed in people exhibiting sundown syndrome (SS). SS was first defined as “disordered cognition, attention, sleep-wake pattern, [and] psychomotor behavior” with a tendency to be more pronounced at night. Many SS cases were found among adults subjected to post-acute and institutionalized care. SS has been attributed to mostly older populations with neurodegenerative diseases, such as AD and Parkinson’s (PD), and is linked to a clinically similar phenomenon colloquially known as “intensive care syndrome (ICS) psychosis” [5]. The clinical characterization of ICS began in the 1950s as reports of extreme behavioral changes and reduced cognitive function steadily increased among patients in intensive care units (ICU). ICS psychosis is displayed by patients who were subjected to regular routines, kept isolated from others, and often in rooms with no windows or little exposure to natural sources of light [6].

One key distinction between SS and ICS for other neurological conditions is that these syndromes have been attributed to older patients with clinically relevant neurodegeneration or patients with no history of mental illness. In other words, these patients maintained some amount of typical neurological function up until they began showing signs of syndrome onset. As such, an examination of CRD in younger patients may provide important insights into how this factor impacts neurological function.

Sleep dysfunction is one of the behavioral manifestations of CRD and is comorbid across neurodevelopmental, affective, and neurodegenerative disorders. While the mechanisms underlying sleep disorders are not entirely understood, many biological components involved are conserved across species. This is illustrated by the characterized rapid eye movement (REM) and non-REM (NREM) sleep stages prevalent in mammals. REM and NREM sleep is associated with distinct brain-metabolic activities and electroencephalography (EEG) signatures [7]. Moreover, changes in sleep ontogeny are shown to parallel periods of synaptogenesis, refinement, and pruning that occur during human brain development indicated by progressive changes in the periodicity of REM and NREM sleep stages. These developmental transitions are associated with alterations in signaling pathways as the brain matures over time [8]. The constitutive components of these signaling pathways coordinate with physiological and environmental cues to mediate the effective transmutation of these system functions. A common component often exploited to examine sleep physiology has been studying melatonin’s behavior (MEL). This endogenously produced hormone is released exclusively at night [9]. Extensive research into the impact of circadian rhythm disruptors (such as light exposure for melatonin production) has helped our understanding of MEL biosynthesis. However, MEL supplementation has shown inconsistent clinical efficacy for treating sleep disturbances, which suggests that more complex processes may be involved in sleep.

## Circadian rhythm disruption in neurodevelopment

### Overlapping symptomatology and pathophysiology across neurodevelopmental disorders

Neurodevelopmental disorders (NDDs) encompass a group of mental health disorders characterized by disruption of typical brain development. Three of the most prevalent NDDs are ADHD, ASD, and TS. Due to the evolving physiological landscape throughout development, mental disorders with early onset may have dynamic effects resulting from damage to other maturing physiological processes. Other defining features, such as distinct behavioral symptoms, are used to establish diagnostic criteria for the American Psychiatric Association’s Diagnostic and Statistical Manual of Mental Disorders (DSM). NDDs are sex-specific, often comorbid, and involve genetic overlaps that suggest commonalities among them.

**Fig. 1:**
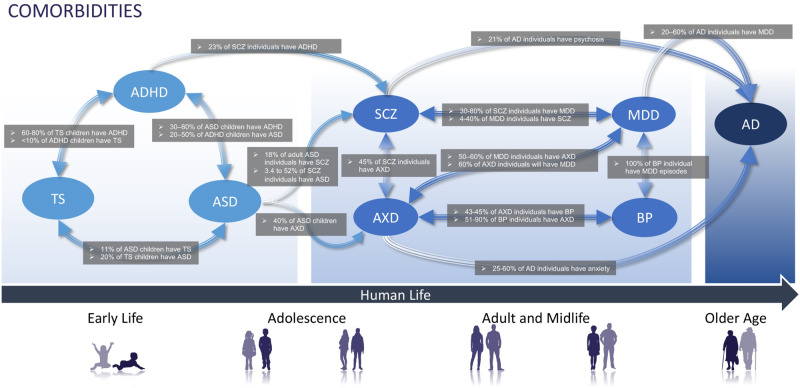
Comorbidities between mental disorder groups. Common characteristics across classically defined mental health disorders were used to determine grouped categories. Highly prevalent comorbidities between mental disorders are related by double-headed arrows illustrating the bifacial nature of each comorbid relationship [140–157]. ADHD Attention deficiency and hyperactivity disorder, ASD autism spectrum disorder, TS Tourette syndrome, BD Bipolar disorder, SCZ schizophrenia spectrum disorder, MDD major depressive disorder, AXD anxiety disorders, AD Alzheimer’s disease.

**Fig. 2:**
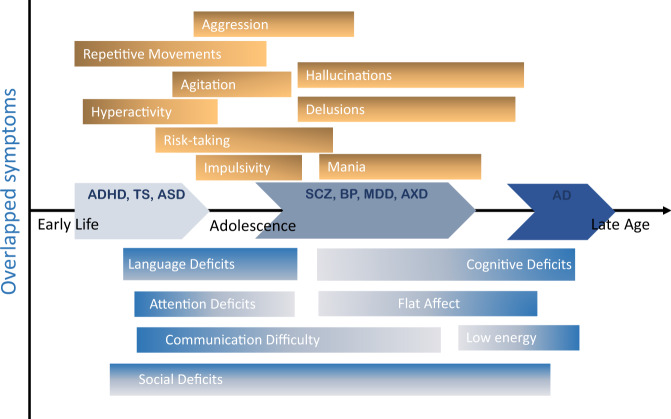
Overlap of symptoms across the major mental disorders with relation to the type of the symptoms and the age onset of disorders. Dominant symptoms respective to age-related mental disorders are represented by respective labeled bars. The typical age-of-onset for each symptom is characterized by the linear start position of each bar, and symptoms have been separated according to classification whereby excessive (surfeit) symptoms are positioned above the horizontal axis and deficits are positioned below the horizontal axis. ADHD Attention deficiency and hyperactivity disorder, ASD autism spectrum disorder, TS Tourette syndrome, BD Bipolar disorder, SCZ schizophrenia spectrum disorder, MDD major depressive disorder, AXD anxiety disorders, AD Alzheimer’s disease.

On average, children with TS receive two comorbid diagnoses in their lifetime, with ADHD being the most common [10] (Figs. 1 & 2). Tics are associated with other NDDs related to impulse control, like ADHD. Moreover, behavioral symptoms related to impulsivity are associated with later age-of-onset disorders, like Parkinson’s disease and affective disorders. This overlapping symptom and its association with dopaminergic dysfunction suggest some dependent pathological mechanisms concerning age.

For example, young adults with TS display social responsiveness deficits [10]. Similarly, social deficits are a hallmark feature of ASD. Characteristic behavioral phenotypic features of ASD include other communication difficulties, and repetitive behaviors [11]. Outbursts and repetitive behaviors relay emotional regulation difficulties [12], and avoidance behaviors are likely due to underlying sensory processing problems [13]. Due to the considerable overlap between ASD and ADHD, studies investigating the connection between sensory processing and inattention reflect the need for a deeper understanding of NDDs’ etiopathogenic roots (Figs. 1 & 2).

Neurobiologically focused research approaches concentrated on specific genetic components and environmental exposures to help identify causative agents or processes underlying ASD and ADHD, and TS. Heritability across these conditions suggests a common genetic link [14]. However, phenotypic expression of any NDD depends on comprehensive effects implicated in single mutations and epistatic interactions among affected genes [15]. Thus, circadian rhythm disruption may impact these epistatic interactions such that phenotypic outcomes for similar genotypes are highly dynamic. A cross-disorder genome-wide association study (GWAS) identified 18 genes significantly related to ADHD, ASD, and TS [16].

### Shared circadian rhythm disruption across neurodevelopmental disorders

A robust link exists between circadian rhythm disruption during pregnancy, induced by maternal stressors, and the development of NDDs [17]. Maternal stressors manifest in different forms, from psychosocial (e.g., maternal mental disorder, lifestyle factors) to physiological (e.g., infection, environmental toxin, and disease). Pregnant mice subjected to chronic circadian disruption gave birth to pups displaying social avoidance behaviors and hyperactivity in adulthood [18]. These results may generalize to human populations, as circadian disruption during pregnancy easily results from shift work, extended work schedules, and prolonged exposure to artificial light [19].

Prenatal stress effects research on the functionality of the suprachiasmatic nucleus (SCN) showed that adult male mice from stressed mothers exhibited altered clock gene expression in the SCN and behavioral phase shifts with impaired stability. Along with central rhythm disruption, exposure to maternal stress had lasting effects on the circadian behavior of the adrenal glands and glucocorticoid (GC) signaling in their adult offspring [17]. As a consequence of maternal stress and associated circadian rhythm disruptions, children are at higher risk of developing NDDs and develop adult circadian rhythm disruption. Indeed, the prevalence of sleep disruptions for NDDs is high; 60% of patients with TS, 70% of patients with ADHD, and 80% with ASD report having comorbid sleep disturbance [20].

Interactions between the circadian CLOCK system and the hypothalamic-pituitary-adrenal (HPA) axis mediate behavioral and physiological adaptations that occur in response to day/night cycles and random stressors. The central CLOCK system describes a highly conserved molecular “clock” which sets internal circadian rhythms according to light/dark inputs into the SCN. The molecular machinery involves regular oscillating activation of a transcriptional loop wherein the heterodimer formed by Clock and Bmal1 stimulates expression of other CLOCK-related proteins, such as Rev–erbα [21]. Mice born to stressed mothers showed reduced mRNA expression levels of negative GC-responsive element (nGRE)-containing clock genes, such as Clock and Rev-erbα, in the adrenal gland and liver. Furthermore, SCN explants from prenatal stress-exposed mice showed altered expression of clock-related proteins (inferred from promoter mPer1 expression), which was associated with impaired synchrony among individual SCN cells in the prenatal stage. Accordingly, it has been suggested that prenatal stress promotes intrinsic defects to the SCN and impacts central rhythm robustness [17]. Additionally, hyperactivity of the HPA axis in pregnant mice in response to prenatal stress is associated with reduced sensitivity to negative feedback mediated by GC signaling in adult mouse offspring [22]. During the gestational stage, mother’s GCs can cross the placenta to play a crucial role in fetal development [23, 24]. GC receptors are expressed in various fetal tissues including the liver, lungs, gut, skeletal muscle, and adipose tissue, where GCs act as essential drivers of normal fetal development and maturation of these organs and tissues, as well as of the timing of their maturation [25–30]. Furthermore, GCs during the gestational stage also induce long-term effects on energy regulation, shaping metabolic plasticity in postnatal life [31, 32]. Mechanistically, GCs during gestation act to regulate gene expression and epigenetic processes, and stimulate cell maturation and differentiation [11, 26, 33, 34]. There is some evidence that the emergence of circadian rhythms occurs only during embryonic cell differentiation, and not in embryonic stem cells (ESCs) or early embryos, and that circadian entrainment may drive functional maturation of stem-cell-derived products [14, 35]. GCs are shown to reset peripheral CLOCKs by influencing the expression of several clock-related genes in peripheral tissues and brain regions (e.g., amygdala) [21]. Accordingly, dysregulated secretion of adrenal cortisol in mice exposed to prenatal stress effectively impacts the rhythmicity of the SCN as well as other physiological processes that rely on circadian rhythm [17].

The HPA axis is the neuroendocrine pathway that regulates GC activity in response to stress [36]. The “stress hormone” cortisol is the primary GC released by the adrenal cortex [37], and abnormal cortisol profiles are consistently associated with NDDs. On the other hand, it is well known that cortisol-related endocrine disorders, such as Addison’s (adrenal gland insufficiency) and Cushing’s (endogenous cortisol excess), are associated with disturbed circadian rhythms [38–40]. Children with ASD have slower cortisol responses when compared with typically developed sex-matched peers [41]. HPA axis dysfunction has long been associated with ASD and was suggested to underlie an increased sensitivity to environmental stressors [42]. Indeed, sustained release of the GC cortisol is connected to hypervigilance, promotion of inflammation, and metabolic dysfunction [37]. A recent meta-analysis revealed that ASD, ADHD, and TS are associated with maternal immune activation, suggesting a convergent pathway in causing fetal neuroinflammation [43]. As many as a third of children with ASD present with abnormal metabolic biomarkers [44]; and ADHD-like behavioral symptoms, including locomotor hyperactivity and inattentiveness, were associated with stress-induced metabolic hypoactivity in prefronto-limbic brain areas [45]. Low evening levels of cortisol are also associated with TS [46]. Thus, it might be possible that the observed comorbid conditions associated with NDDs are related to dysregulation of GC activity and mediated by maternal stress exposure. Because of the interdependent relationship between the circadian CLOCK system and the HPA axis, studies of prenatal stress with respect to circadian rhythm disruption may help elucidate prevention strategies to minimize fetal exposure. Animal models of metabolic dysfunction during pregnancy (a physiologically-based maternal stressor) use defective placental insulin receptor (InsR) signaling to exhibit sex-specific alterations in HPA axis stress responses and changes in gene expression reflecting potential shifts in serotonin (5-HT) homeostasis and mitochondrial function in mice [47].

A significant association of two single nucleotide polymorphisms (SNPs) in PER1 and two in NPAS2, and a decreased CLOCK gene expression were observed in individuals with ASD [48, 49]. Further, heterozygous coding variations in the CRY1 were reported in families with combined ADHD and insomnia [50].

Aside from GC-mediated signaling, serotonin is also an essential stress-related signaling neurotransmitter. This neurotransmitter readily crosses the placental barrier and influences fetal brain development, particularly with respect to neuronal migration and proliferation [51]. In relation to circadian rhythmicity, serotonin is also the precursor to MEL. MEL, or N-acetyl-5-methoxytryptamine, is a hormone primarily released at night by the pineal gland and derived from serotonin via two enzymatic reactions [52]. In the presence of light, MEL production is inhibited [53]. Studies show that the SCN regulates the synthesis of pineal MEL. At the same time, extrapineal MEL is spread throughout the body—namely, the placenta and uterus, consistent with its role as a neuroprotective agent for the fetus [54]. As a vital antioxidant, MEL engages in free radical scavenging, regulates neurotrophic factors and cytokine production, and manages the expression of genes involved in neuronal proliferation, differentiation, structure, and metabolism. In the context of NDDs, there is a delicate balance in utero between reactive oxygen species (ROS) and antioxidants, such as MEL, to maintain homeostasis. As such, disruptions in MEL concentration may increase oxidative stress burden during pregnancy, which may be significant enough that the fetus incurs some degree of oxidative damage. Prenatal conditions associated with increased oxidative stress include diabetes, obesity, preeclampsia, and smoking [55]. Population statistics show that these prenatal conditions are also highly correlated with NDD diagnoses in children. These findings suggest that dysregulation of oxidative metabolisms, such as decreased MEL antioxidant capacity due to maternal stress, may be related to greater NDD susceptibility in children.

Light controls MEL’s expression levels, and its circulation entrains peripheral organs via interactions with molecular clock mechanisms [4]. Entrainment of circadian rhythms results in patterns of oscillating photosensitive periods (photoperiod) and defines regular fluctuations in physiological activity with respect to time spent sleeping. Fetal exposure to maternal stress and maternal circadian rhythm disruption can alter these rhythms in early life. Studies investigating the effects of constant light exposure during pregnancy reveal that this condition suppresses the emergence of normal MEL and body temperature rhythms in offspring following birth [19]. These experiments were designed to simulate “shift-work,” as rotating night shift work is linked to circadian rhythm disruption in expecting mothers and their children [56]. In nonhuman primates, the fetal biological clock is responsive to maternal entraining signals and is observed to begin oscillating by the third trimester of pregnancy [57]. This finding coincides with the finding that, during normal human pregnancy, the expression of enzymes involved in MEL biosynthesis peaks during the third trimester [58]. MEL receptors are present in the fetal brain, though production of MEL begins postnatally. Therefore, offspring are entirely dependent on maternal MEL secretion [59]. Since MEL can readily enter the fetal compartment from maternal circulation, changes in maternal serum MEL may impact developmental processes that rely on it for signaling. In a study examining the effects of maternal MEL, investigators found that MEL is neuroprotective in utero and protects against tissue damage related to fetal inflammation [60].

Studies into maternal MEL deprivation have also shown sex-specific neurobehavioral developmental differences, wherein male offspring exhibited hippocampal-dependent spatial reference and working memory deficits [61]. Hippocampal-related cognitive deficits are associated with several NDDs, such as AD and ASD, and hippocampal atypical morphological differences correlate with TS and ADHD [62]. These findings suggest a possible link between maternal MEL dysregulation and regionally specific cognitive deficits in offspring. ASD is consistently linked to reduced MEL levels in the evening, and abnormal MEL production rhythms have been reported in ADHD [19].

## Circadian rhythm disruption in adolescence through adulthood

### Overlapping symptomatology and pathophysiology of adolescence and midlife mental disorders

Some psychiatric disorders, such as SCZ, anxiety, eating disorders, depression, and bipolar disorder, typically emerge during adolescence or early adulthood. Though manifested by seemingly distinct behavioral phenotypes, symptoms can overlap across various mental health disorders. Schizophrenia (SCZ) is a heterogeneous set of symptoms that often appear around early adulthood [63]. SCZ onset occurs in adolescence and young adulthood for both genders and peaks around 40 years of age. Though prevalence tends to decline with age [64], childhood-onset schizophrenia (COS) is possible. ASD was once identified as childhood-onset SCZ. While COS is extremely rare, affecting only 1 in 10,000—30,000 children, ASD remains one of the most prevalent NDDs [65]. The criteria for SCZ diagnosis specify that one of the symptoms has to be either delusional, hallucinatory, or disorganized thinking lasting a minimum of one month. SCZ is progressive; individuals with SCZ suffer a substantial decrease in quality of life due to psychotic episodes, disordered thoughts, memory deficits, and a loss of interest in once enjoyable activities.

Bipolar disorder (BD), an affective disorder, is characterized by recurrent manic or hypomanic episodes that may alternate with depressive episodes [66]. Mania is an abnormally elevated or irritable mood associated with daily increases in activity or energy for at least one week. Mood disturbances may be severe enough to impair social and occupational responsibilities. In 75% of manic episodes, psychotic symptoms like delusions and hallucinations occur [66], serving as one of many details that blur the boundaries between BD and SCZ (Figs. 1 & 2).

Major depressive disorder (MDD) shares symptoms with major depressive episodes (MDE) episodes of BD, with a critical difference in that MDE can co-occur with manic episodes. As a result, BD is often misdiagnosed as MDD [66]; differentiating between major depressive episodes (MDE) of BD and symptoms of MDD is particularly complicated (Figs. 1 & 2) as the duration of symptoms qualifying a BD diagnosis has no biological basis. Survey responses from different age-at-onset groups revealed that the mildest, least pervasive, dysfunctional, and recurring forms of MDD were associated with patients with later ages of onset. In contrast, childhood and adolescent-onset MDD correlates with much more significant functional impairment [67].

Anxiety disorders (AXD) include panic disorder, generalized anxiety disorder, social anxiety disorder, obsessive-compulsive disorder, and phobias. AXD exhibits onsets ranging from early adolescence to young adulthood [68]. Moreover, though AXD expression is sex-specific, evidence suggests no sex dependence regarding the age of onset. These findings align with theories positing a sensitive period wherein females are more susceptible than males to disorders characterized by heightened emotionality and social vigilance during adolescence [69]. Regarding later-onset AXD, studies have consistently shown AXD to be a reliable predictor of cognitive decline in those presenting with mild cognitive impairment. Indeed, it has been suggested and corroborated by study results that midlife AXD itself may be a risk factor for late-life dementia [70].

The etiology of SCZ and other affective disorders remains mostly unknown. The Dopamine Hypothesis first emerged about pharmacological evidence—specifically, the ability of dopamine antagonists to reverse psychotic symptoms [63]. Recent studies support the neurodevelopmental hypothesis suggesting that disruption of brain development in early life causes psychosis in adulthood. Thus, the etiology of SCZ develops from complex interactions between genetic and environmental factors during critical periods of development [71, 72]. Researchers suggest that neuroinflammation and breakdown of the blood-brain barrier (BBB) contribute to the cognitive and behavioral symptoms in SCZ [73]. Moreover, studies have found BBB hyperpermeability correlated with other psychiatric disorders, namely MDD and BD [73].

As with SCZ, the causes of BD and MDD are not fully understood; however, strong evidence suggests that BDNF may play a role in the onset of MDD and BD since it is abundant in brain regions implicated in affective disorders. The serum BDNF decreases in BD patients during manic or depressive episodes compared to euthymic patients [74]. Further, the severity of mania and depression in BD patients seems to inversely correlate with BDNF levels [75]. The stress-BDNF theory of MDD postulates that BDNF that both acute and chronic stress decrease BDNF levels, which may contribute to brain atrophy and cell loss in the brain regions associated with affective disorders, including the hippocampus.

Research suggests that an anxious temperament and behavioral inhibition in children and adolescents are heritable early-life risk factors for developing AXD, other affective disorders, and comorbid substance abuse [76]. The amygdala plays a vital role in fear, aggression expression, and memories; thus, it is implicated in several brain disorders, particularly affective disorders. Neuroimaging studies show increased activity of emotion-related regions such as the amygdala [77, 78].

### Shared circadian rhythm disruption in adolescence and midlife mental disorders

Recent studies of the connection between chronotype and psychopathology are both sex- and age-dependent [79]. Chronotype describes the intrinsic circadian variation across individuals, which informs their natural sleep/wake behaviors in terms of a single day-night cycle. Identifying different chronotypes may provide evidence of differences in circadian rhythmicity, especially in cases of CRD. Generally, subgroups are defined according to their preference to be awake during morning hours (M-type) or evening (E-type). Though light exposure and sleep quality play significant roles in establishing waking behaviors, growing evidence suggests that E-types are more correlated with adverse psychological outcomes, including depressive and anxiety symptoms and behavioral dysregulation [80]. In young adults, the E-type is linked to aggression and hostile behaviors in men, while it is associated with aggression and antisocial behavior in childhood through adolescence [80]. With respect to adolescents and young adults diagnosed with common mental disorders such as AXD and MDD, E-type females are significantly more affected by one or more psychiatric disorders [80, 81].

Furthermore, E-types of both sexes have been associated with novelty-seeking behavior, irritability, and cyclothymic fluctuations [80]. The chances of CRD are substantially enhanced due to the increasing amount of time spent in front of screens and other sources of artificial light. Those with an inherent predisposition for the E-type may be at greater risk of adverse psychological consequences related to an increased disparity between one’s endogenous circadian rhythm and the 24-hour light/dark cycle. Disruptions of physiological mechanisms that rely on effective circadian synchronization, such as appetite control, may exacerbate other interrelated processes and manifest behaviorally as aggression, irritability, or anxiety. Desynchronization for adolescents with a tendency toward evening-ness, particularly women, may impact sleep behaviors. Proper sleep hygiene and good sleep quality are associated with characteristic brain activation in areas crucial for attention and bodily awareness. Performing tasks at non-optimal times of the day may result in cognitive impairments resulting in, for instance, slower reaction times and higher error rates [80, 81].

Patients with BD or MDD often suffer from sleep disruption. During the manic episode in bipolar I, 69–99% of patients report a reduced need for sleep [82]. Robillard et al. also showed that during MDE, 38%-70% of bipolar patients reported hypersomnia and severe insomnia. Interestingly, evidence indicates that BDNF induces sleep disorder and spontaneous wakefulness in animals [83]; this, in turn, affects neuron survival, plasticity, development, and functions, all of which are related to BD and MDD.

Sleep deprivation has many negative consequences like disrupted mood and cognitive dysfunction, impaired motor performance, irritability, and emotional volatility [84–86]. Sleep deprivation also affects other brain functions; 24-h continuous wakefulness reduces glucose metabolism in the prefrontal cortex [87]. Evidence has revealed a complete circular relationship between stress and sleep disorders, wherein stress provokes sleep disorders, and disturbed sleep provokes stress. Further, studies showed that sleep disorder is an early symptom (prodrome) before the manic or depressive episode [88]. Imaging studies have shown that brains of healthy individuals deprived of sleep mimic certain pathological psychiatric patterns and that sleep patterns predict the co-occurrence of psychiatric symptoms [89–92].

Synaptic plasticity is affected by light and circadian rhythmicity, and the expression of plasticity-associated synaptic proteins such as Shank proteins follow circadian-like oscillations [93]. Genes encoding Shank3 proteins are associated with NDDs, SCZ, and AD, and CRD in mice causes alterations in hippocampal Shank3 expression levels, paired with substantial disruptions to plasma MEL levels in mice exposed to CRD [93]. Neurexins, cell adhesion molecules involved in synaptogenesis and synaptic transmission, control sleep quality and sleep homeostasis by mediating αβ neurons’ synaptic transmission [94]. Copy number variants (CNVs) of neurexin genes have been linked to ASD and SCZ [95]. These findings provide evidence for a bidirectional relationship between CRD and neuroplasticity mechanisms implicated in mental disorders.

## Circadian rhythm disruption in late age

### Overlapping symptomatology and pathophysiology of aging-related mental disorders

Aging-related neurodegenerative disorders, like AD and Parkinson’s disease (PD), are progressive in that behavioral and cognitive impairments increase in severity with age. Impaired functions include memory, language comprehension, attentional control, decision-making, and mood and personality changes over time. Old age is the most significant risk factor for AD; hence, women are more susceptible to ADs since they live longer than men on average [96].

AD progresses through three main stages: pre-symptomatic, mild cognitive impairment, and marked dementia. Patients’ sleep becomes fragmented, and their visuospatial abilities become impaired as they lose their regular circadian sleep-wake pattern [97]. The literature suggests that symptoms seen in age-related neurodegeneration may be mediated by the same neural circuitry [98]. Susceptible genotypes are at an increased risk of developing a mental health disorder through epigenetic mechanisms related to age and severity of pathological consequences.

AD pathogenesis implicates abnormal accumulation of extracellular aggregates of beta-amyloid (Aβ) plaques and intracellular aggregates of neurofibrillary tangles (NFTs) [99]. Aβ pathogenesis starts with amyloid precursor protein (APP), a plasma membrane protein. APP is aberrantly cleaved by β-secretases (BACE1 enzyme), and γ-secretases (complex of presenilin 1 or 2, nicastrin, APH-1) produces insoluble Aβ fibrils. Oligomerized Aβ diffuses into synaptic clefts and interferes with synaptic signaling [100–102]. BDNF levels become deficient in AD. Animal model studies show that BDNF treatment rescued the expression of perturbed genes due to mutant APP expression in both the entorhinal cortex and the hippocampus. This results in learning and memory improvements in hippocampus-dependent tasks [103].

### Shared circadian rhythm disruption in aging-related disorders

Decreased sleep duration and/or fragmentation and circadian alterations are often observed in early AD. CSF Aβ concentrations show a daytime fluctuation in humans, increasing during the day and decreasing at night [104]. In animal studies, sleep to helps regulate the clearance of toxic metabolites from the adult brain. Therefore, sleep disturbances may impair the removal of neurotoxic waste products accumulated during the wake period, such as Aβ from the brain [105]. Sleep-wake rhythmicity is found to affect the pathology of Aβ in the brain. Further, sleep deprivation or an increase in wakefulness caused by rescuing the function of orexin neurons in APP/PS1 mice lacking orexin increased the amount of Aβ pathology in the brain, suggesting a modulatory role of orexin, especially with respect to Aβ pathology in the brain [106]. Furthermore, sleep fragmentation has been shown to increase the incidence of AD and cognitive decline in adults, associated with Aβ accumulation. This decreases the quality of wake/sleep states and may affect hippocampus-dependent memory, resulting in progressive memory impairment in mouse models and AD patients [107].

Additionally, SNPs in CLOCK and BMAL1 genes are associated with higher susceptibility to developing AD disease [108, 109]. Since BMAL1 regulates oxidative stress, glial activation, and neurodegeneration, the pathogenic impact of Aβ on BMAL1 might mediate an increase in the inflammatory response and neurodegeneration, resulting in neurotoxicity [110]. Indeed, sleep deprivation is found to enhance the brain’s inflammatory state by activating glial cell pathways, which are essential in memory and synaptic plasticity, as well as by acting on cytokines and other proinflammatory markers [111]. The enhanced inflammatory state might render the hippocampus more vulnerable to Aß-driven neurotoxicity [112]. The circadian clock positive-limb transcriptional complex (BMAL1:CLOCK/NPAS2), which regulates the transcription of many circadian and non-circadian genes relevant to neuronal function, has a vital role in neuronal redox homeostasis and neurodegeneration protection [113]. Inflammation increases the Aβ burden and is believed to result in AD disease pathogenesis [114].

MEL production decreases with age, with more severe disruptions in patients with age-related neurodegenerative disorders such as AD [115]. Across the entire lifespan, MEL levels peak at 1–3 years of age, and by adolescence through early adulthood (15–20 years old), there is already an 80% reduction in MEL levels, which continue to decline into old age [116]. Thus, MEL’s role as a powerful free radical scavenger and potent antioxidant becomes more important as the physical body ages. Through various mechanisms, MEL attenuates damaging effects of systemic immune activation (inflammaging), which increase with age.

## Sex context of circadian rhythm disruption in mental disorders

Sex-specific structural and functional differences exist within the brain in regions relevant to circadian systems. Similarly, sex-dependent features, like sexually dimorphic physiology are linked to CRD with respect to male-dominant brain disorders. Thus, potential sex-age interactions related to these disorders may have a functional impact on circadian rhythmicity. Sex hormones that influence circadian rhythmicity mediate physiological changes associated with adolescence and puberty. Sex differences in circadian mechanisms may therefore underlie sex-dependent consequences of CRD with respect to mental health disorders known to have a typical onset during this stage in life. Studies of the effects of “shift work” showed sex-dependent differences in feeding behaviors and related hormones upon circadian misalignment.

The SCN is sexually dimorphic and enriched with estrogen receptors [117]. Recent studies have shown that daytime activation of the SCN was significantly higher in male mice [118]. Consequently, innate sex differences in estrogen receptor expression within the SCN and sex- and age-dependent changes to circulating estradiol contribute to receptor patterning and estrogenic signaling capacity. Circulating estrogens relating to estrogen receptor availability may create intrinsic sex-specific and age-dependent differences. Moreover, circadian clock genes and estrogen receptor signaling functions bidirectionally. Because estrogenic signaling is known to mediate many developmental and operational processes in the brain, it may be essential to research the functional relationship between estrogenic signaling with respect to circadian rhythms.

Sex differences exist in the serotonergic inputs to the SCN. Both ESR1 and ESR2 are expressed in the medial and dorsal raphe nuclei. In contrast, only androgen receptors (ARs) are present in the dorsal raphe nuclei of male rats and mice [119]. Circadian clock gene and estrogen receptor signaling appear to function in a bidirectional relationship [117]. Considering the sex-age interactions with the SCN, one might propose that sensitivity to light is impacted in males upon experiencing pubertal surges in testosterone. Similarly, women may lose estrogenic potency at the SCN after menopause, when estrogen-related signaling drastically diminishes.

Studies on rats showed that the diurnal activity of MEL-related enzymatic profiles in castrated male adult rats followed similar oscillatory activity to female animals and that testosterone substitution in male castrated rats resulted in a pattern, which resembled that of adult male controls—a pattern that appeared to develop during the infantile period of life [120]. These findings reveal intrinsic sex-dependent divergences in circadian entrainment in rodents, which suggests the existence of similar biological factors in humans.

BDNF has consistently been linked to several mental health disorders covered in this review, with functional roles attributed to genes that can be connected to CRD. Studies have generally demonstrated downregulation of BDNF gene expression in patients with SCZ and mood disorders [121], while increased BDNF serum levels have been reported in ASD children and ADHD boys. In contrast, ADHD girls were found to have lower BDNF levels than the control girls [122–124]. The elevated levels of BDNF in ASD and ADHD were hypothesized to present a compensatory mechanism to facilitate neurodevelopment among children whose brain development is delayed. Early life stress causes a female-specific increase of BDNF expression in the amygdala [125]. Plasma BDNF levels were found to follow a diurnal rhythm in men, with maximal and minimal concentrations occurring during early light and dark hours respectively. On the other hand, in women BDNF plasma levels were more temporally stable regardless of the menstrual phase [126]. Further, the relationship between BDNF polymorphisms and cortisol activity has exhibited significant gene-by-sex interactions. High blood cortisol levels in response to psychosocial stress showed genotypic effects among females only [127].

## Concluding remarks and implications for future research

**Fig. 3:**
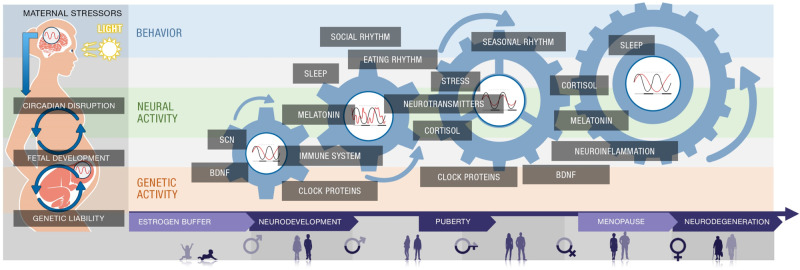
A model for the impact of CRD on genetic, neurological, and behavioral factors over lifespan. The impact of CRD on brain disorders is pervasive and dynamic. Starting from the fetal stage through early life, the effects of maternal CRD (e.g., resultant from asynchronous light exposure) on development can impact genetic and neural activity with respect to individual genetic liability. During this period, estrogen is proposed to act as a buffer against neuropathological mechanisms. However, hormonal changes throughout life will eventually curb these protective effects. Moreover, behavioral factors play an increasingly significant role in directing the repercussions of CRD with age. The consequences of the interplay between circadian-sensitive genetic (red bar), neural (green bar), and behavioral (blue bar) factors through late age are profound and complex, as dynamic interactions over time result in bidirectional relationships among all circadian-sensitive factors. The labeled factors in each bar have been consistently associated with neurodevelopmental disorders (NDDs), adolescence and midlife psychiatric disorders, and aging-related neurodegenerative disorders. The ones highlighted in the figure do not represent a complete list.

The evidence presented in this perspective suggests that CRD is a common underlying p-factor that bridges across mental disorders. We propose *circadian rhythms* disruption as a factor for novel theories and experimental approaches for bridging various disorders, which takes into account the effect of age and sex (Fig. 3). In particular, because circadian rhythms are intrinsically sensitive to light-dark cues, they can be easily disrupted by asynchronous (or artificial) light exposures at night, and this disruptive capacity appears to be sex-dependent and changes with age. Perturbations during the early life of circadian entrainment can have long-lasting behavioral effects on offspring [18]. One component of chronic circadian disruption is dysregulated MEL production during pregnancy, due to physiological or emotional maternal stress. This concept is supported by the finding that MEL supplementation and bright light therapy are most commonly used to treat circadian disruption in patients with ADHD and ASD [128, 129]. Therefore, there might be clinical implications for pregnant women and their unborn children exposed to chronic stress and CRD.

**Fig. 4:**
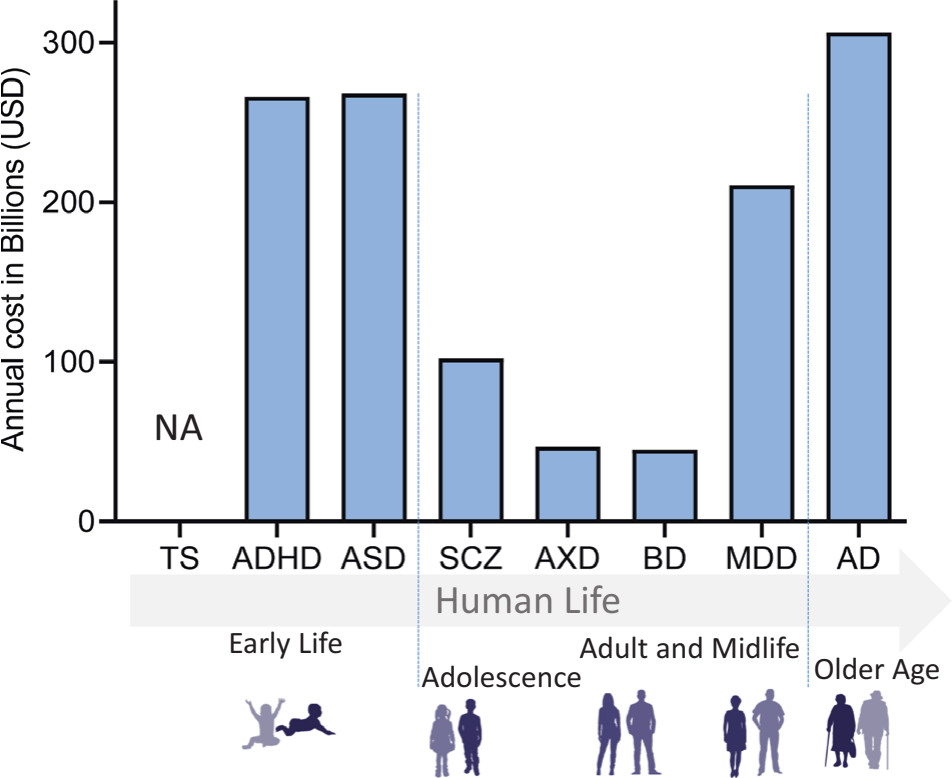
Annual costs of mental disorders. The annual cost of mental health disorders in the U.S. per disorder (in USD). Mental health disorders are grouped according to typical age-of-onset (Data sources: [158–162]). ADHD Attention deficiency and hyperactivity disorder, ASD autism spectrum disorder, TS Tourette syndrome, BD Bipolar disorder, SCZ schizophrenia spectrum disorder, MDD major depressive disorder, AXD anxiety disorders, AD Alzheimer’s disease. NA Not available (https://www.cdc.gov/ncbddd/tourette/bridgingthegap.html).

**Fig. 5:**
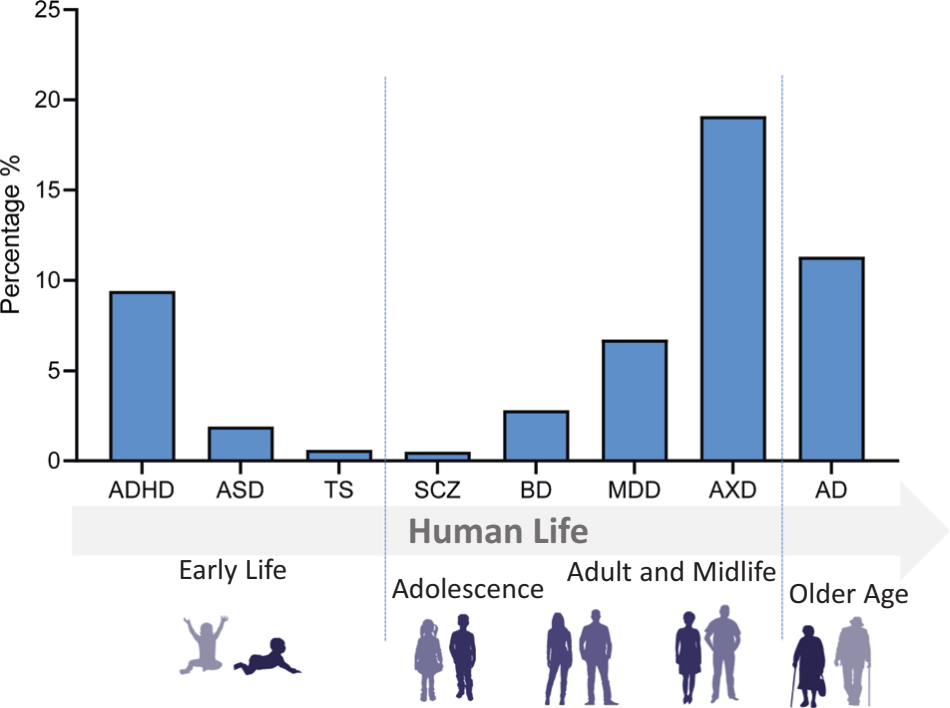
Prevalence of mental disorders related by age. The prevalence of mental health disorders as a percentage of age-matched. Mental disorders are grouped according to typical age of onset. Data sources: [162–168]. ADHD: Attention deficiency and hyperactivity disorder, ASD autism spectrum disorder, TS Tourette syndrome, BD Bipolar disorder, SCZ schizophrenia spectrum disorder, MDD major depressive disorder, AXD anxiety disorders, AD Alzheimer’s disease.

**Fig. 6:**
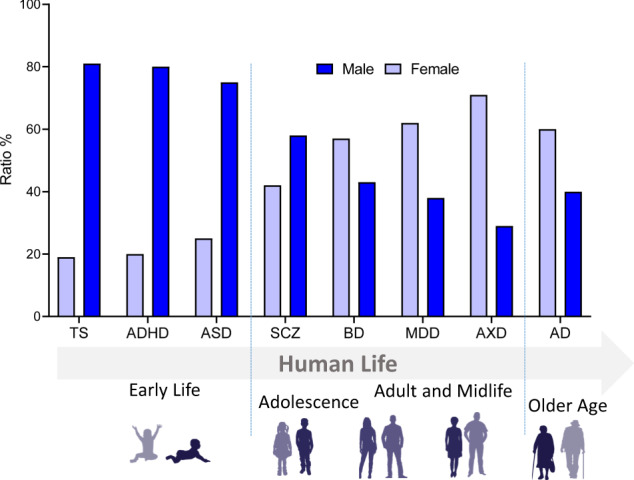
Sex-dependent prevalence of mental disorders as related to age. Mental health disorder prevalence in males and females illustrated as ratios of the total affected population per disorder. Mental disorders are grouped according to typical age of onset. Data sources: references [169, 140, 170–175]. ADHD Attention deficiency and hyperactivity disorder, ASD autism spectrum disorder, TS Tourette syndrome, BD Bipolar disorder, SCZ schizophrenia spectrum disorder, MDD major depressive disorder, AXD anxiety disorders, AD Alzheimer’s disease.

We argue also that the CRD p-factor hypothesis can be generalized to other mental health disorders, such as OCD, eating disorders such as anorexia nervosa, bulimia nervosa and food addiction and PD. For example, a form of CRD (REM sleep disorder) has not only been shown to precede the development of PD in some patients, but CRD is well documented to occur as the disease progresses. Many PD patients suffer of various CRD symptoms, such as increased sleep latency, reduced sleep efficiency, and reduced REM sleep, and they exhibit an elevation of serum cortisol levels and reduced circulating melatonin levels [130].

Even though our perspective demonstrates that CRD cuts across all disorders, the reported evidence results largely from observations made at a level that does not allow inferring causal relationships. Therefore, it remains essential to identify potentially causative relationships between CRD and mental health disorders. These relationships may provide insight into the unresolved etiologies of these disorders and inspire novel therapeutic approaches. One approach is to cross-match known genetic risks and morphological changes across the disorders and then correlate these matches to signs of CRD to find potential causative relationships worth exploring. Another powerful approach is to conduct detailed, high-throughput, circadian omic (transcriptomic, metabolomic) studies in healthy and diseased subjects. While this approach can only be carried in a limited way in humans (e.g. using serum), it could be applied on a large scale to animal models [131, 132]. In particular, across the mouse models of the various disorders, it could be applied in a systematic way with respect to age, sex, brain areas, and so forth to investigate circadian molecular rhythmicity before and during the disease progression, and ultimately help identify potential biomarkers, causal relationships, and novel therapeutic targets and avenues.

Finally, it is likely that other P-factors are also involved. A likely candidate could be disruptions at different levels of the brain gut microbiome system, as demonstrated by a series of preclinical and clinical studies showing alterations not only in the interactions with circadian rhythms [133, 134] but also in the pathophysiology of several neurodevelopmental disorders [135], PD [136], and ASD [137]. Another candidate could emerge from the interactions between nutrition, cellular metabolism, and epigenetics in neurodevelopmental and other mental disorders [138, 139].
